# Illustrations of the Botany and Other Branches of the Natural History of the Himalayan Mountains, and of the Flora of Cashmere

**Published:** 1836-01

**Authors:** 


Art. XII.—*Illustrations of the Botany and other Branches of the Natural History of the Himalayan Mountains, and of the Flora of Cashmere.* By J. Forbes Royle, Esq., f.l.s., &c. *London. August,* 1835. *Part VIII.* Folio.

As only a single number of this splendid book has come into our possession, since we commenced our critical labours, we must put off to a future occasion such a review of the whole of the Parts published, as the importance of the work claims at our hands. We must, however, say of the present Part that the plates, eleven in number, nine of botanical and two of zoological subjects, are extremely beautiful, being very finely coloured, and the representations clearly possessing that *naturalness* of character which enables us almost to say that a portrait is *like,* even although unacquainted with the original. The text is rich in original details, as well as in elaborate scientific researches; and every thing bearing on the Materia Medica, whether as acknowledged in the schools or as existing only in the unwritten dispensatories of the natives of the country, is studiously noticed. Mr. Forbes Royle, amid his botanical science, seems always to keep in view his own profession.

---

Art. XIII.—*The British Medical Almanack, and Supplement.* 1836. *London.* Small 8vo. Pp. 160.

